# Deconvoluting AMPK dynamics

**DOI:** 10.18632/oncotarget.5447

**Published:** 2015-09-01

**Authors:** Takafumi Miyamoto, Elmer Rho, Takanari Inoue

**Affiliations:** Department of Cell Biology, Center for Cell Dynamics, Institute for Basic Biomedical Sciences, Johns Hopkins University, School of Medicine, Baltimore, MD, USA

**Keywords:** signaling, biosensors, compartmentalization, FRET, diabetes

AMP-activated protein kinase (AMPK) is an evolutionarily conserved heterotrimeric complex composed of α (catalytic α1 and α2) and β/γ (regulatory β1, β2, γ1, γ2, and γ3) subunits and serves as a gatekeeper of energy metabolism by responding to metabolic needs of a cell. Recent findings implicated the enzyme in a broad range of cellular functions, including cell growth, autophagy, polarity, transcription, as well as metabolism [1]. AMPK has thus emerged as a potent target for treating diseases such as cancer and diabetes.

Previously, Cantley and colleagues have reported findings regarding compartmentalized signaling of AMPK using a FRET-based AMPK biosensor [2]. AMPK biosensors localized to intracellular compartments such as nucleus and cytosol have revealed that glycolysis inhibitor, 2-deoxyglucose (2-DG), increased AMPK activity in the cytosol but not in the nucleus. In contrast, ionomycin-induced influx of calcium ions caused AMPK activation in both regions. AMPK fulfills this role likely by dynamically adjusting its activity at individual compartments. These observations implied that AMPK is part of a broader signaling network involving other subcellular localizations.

We recently improved the dynamic range of a series of organelle-specific AMPK biosensors [3], [4]. With the improved properties of the biosensor, we were able to visualize AMPK activity at many of the major subcellular compartments in real time. We then compared these activity profiles between AMPK-null mouse embryonic fibroblasts (5-DKO :MEFs) and genetically matched wildtype MEFs (5-WT MEFs). Under a nutrient-rich condition, AMPK exhibited various degrees of activation across the different subcellular compartments. In general, AMPK activity at the organellar membrane was significantly higher than in the cytosol or the nucleus. A constitutively active form of AMPK increased in the cytosol and nucleus but was less effective at other organellar membranes. This was further corroborated by fractionation assays revealing that endogenous AMPK was mainly localized at the organellar membranes rather than the cytosol. The emichment of AMPK in non-cytosolic compartments can be a possible mechanism by which the activation threshold is lowered for AMPK-mediated acute responses.

To further examine the compartmentalized AMPK signaling, we measured AMPK activity at each organelle under nutrient-limited conditions triggered either by 2-DG treatment or glucose starvation. As expected, the activity of AMPK was versatile across different compartments to glycolytic perturbations. This suggests that the stimuli were independently processed across the compartments despite similar physiological outcomes. Furthermore, a constituent of the AMPK complexes determined their localization. AMPK complexes that solely contained al subunit tended to favor the plasma membrane, Golgi apparatus, and lysosome; complexes containing either αl or α2 subunit distributed the enzyme equally across the cytosol, endoplasmic reticulum, and mitochondria.

We next developed a genetically encoded molecular inhibitor based on AMPK peptide inhibitor (AIP). By targeting the AIPs to individual compartments, we achieved inhibition of AMPK activity, for example, at the mitochondria or the Golgi apparatus without affecting activity in cytosol. In addition, we gained control over the compartment in which AMPK inhibition took place by rapid recruitment of AIP to the organelle using a chemically-inducible dimerization technique [6]. One noteworthy finding was that prolonged perturbation of AMPK signaling at the mitochondria in WT MEFs caused metabolic reprogramming by which intracellular ATP increased relative to that observed in DKO :MEFs. This suggests that localized perturbation is sufficient for inhibiting specific aspects of AMPK signaling.

In the study presented here, we revealed the intricacy of compartmentalized AMPK regulation in living cells by developing and implementing genetically encoded organelle-specific molecular tools for AMPK. We unambiguously demonstrated that AMPK activity is compartmentalized. Possible future studies could explore how cells achieve spatio temporally precise AMPK activity and identify the molecules that determines AMPK activity under conditions beyond nutrient levels. Understanding the molecular mechanisms that underlie AMPK regulation could provide insights into the development of new therapeutic strategies for AMPK-related diseases.
